# Disruption of Hox9,10,11 function results in cellular level lineage infidelity in the kidney

**DOI:** 10.1038/s41598-018-24782-5

**Published:** 2018-04-20

**Authors:** Keri A. Drake, Mike Adam, Robert Mahoney, S. Steven Potter

**Affiliations:** 1 Division of Developmental Biology, Cincinnati Children’s Hospital Medical Center, Cincinnati, OH 45229 USA; 2 Division of Nephrology and Hypertension, Cincinnati Children’s Hospital Medical Center, Cincinnati, OH 45229 USA; 3 Division of Pediatric Nephrology, University of Texas Southwestern Medical Center, Dallas, TX 75390 USA

## Abstract

Hox genes are important regulators of development. The 39 mammalian Hox genes have considerable functional overlap, greatly confounding their study. In this report, we generated mice with multiple combinations of paralogous and flanking Abd-B Hox gene mutations to investigate functional redundancies in kidney development. The resulting mice developed a number of kidney abnormalities, including hypoplasia, agenesis, and severe cysts, with distinct Hox functions observed in early metanephric kidney formation and nephron progenitor maintenance. Most surprising, however, was that extensive removal of Hox shared function in these kidneys resulted in cellular level lineage infidelity. Strikingly, mutant nephron tubules consisted of intermixed cells with proximal tubule, loop of Henle, and collecting duct identities, with some single cells expressing markers associated with more than one nephron segment. These results indicate that Hox genes are required for proper lineage selection/maintenance and full repression of genes involved in cell fate restriction in the developing kidney.

## Introduction

Hox genes encode highly conserved transcription factors critical for patterning the developing embryo^1^. Hox gene mutation can result in dramatic homeotic transformations in *Drosophila*^2^. For example, the *Antennapedia* mutation causes legs to form on the head in place of antennae^3^. It has been proposed that segment identity can be defined during development by a Hox code^4^, with the specific combination of Hox genes expressed deciding the developmental destinies of the group of cells within a segment.

The Hox gene network in mammals is highly functionally redundant. Mammals have a total of 39 Hox genes arranged in four clusters with 13 paralogous groups. In mammals the axial body segments also show identity transformations in response to Hox mutations, with removal of multiple members of a paralogous group giving synergistic severity phenotypes^5–7^. Hox mutations or ectopic expression can also lead to transformations of hindbrain rhombomeres^8,9^, as well as partial homeotic transformations of reproductive tract segments^10,11^.

While paralogous Hox genes show the greatest functional overlap, there is also extensive evidence indicating that Hox genes near each other on a single cluster are partially redundant. Such flanking Hox genes often show great similarities in their homeodomain amino acid sequences, and Homeobox swap experiments show partial functional equivalence for flanking Hox genes^12,13^. In addition, *Hoxa*10/*Hoxa11* trans-heterozygotes show a synergistic phenotype^14^, and double mutation of the flanking paralog *Hoxa10* and *Hoxd11* genes shows their functional overlap^15^.

Almost all Hox genes show expression during kidney development^16^, yet previous studies have primarily detected kidney development abnormalities only in mice with multiple mutations in either the *Hox10* or *Hox11* paralogs^17–20^. Homozygous mutation of *Hoxa11*^11^ or *Hoxd11*^21^ alone gives normal kidneys, while simultaneous mutation of both genes results in dramatically hypoplastic kidneys^17,18^. Further mutation of all three Hox11 paralogs completely blocks an initial stage of kidney formation, the outgrowth of the ureteric bud from the nephric duct^19^. Hox11 genes are key regulators of SIX2 and GDNF expression^19^, showing that they can function at multiple levels of developmental programs and not just in segment identity determination. Hox10 genes are also required for normal formation of the stromal compartment^20^.

The data suggest, therefore, that the mutation of novel combinations of paralogous and flanking Hox genes could reveal Hox functions in kidney development previously concealed by redundancy. Given this, there are a number of considerations. One approach would be to create LoxP generated deletions of groups of flanking Hox genes. A drawback, however, is the consequent removal of intergenic regional shared enhancers, resulting in ectopic expression of remaining Hox genes, which can significantly confound interpretation^22^. An extreme version of this approach is to create deletions of entire Hox clusters. Surprisingly, however, the deletion of a complete Hox cluster can result in a phenotype that is milder than mutation of a single Hox gene within that cluster^23^. This could be the result of cross regulatory interactions between Hox clusters, such that removal of one cluster results in compensatory elevated expression of others.

Another important consideration is that removal of all Hox11 paralog function blocks very early kidney development, resulting in no subsequent kidney development for investigation. Therefore, the combinations of Hox mutations generated must include at least some minimal measure of Hox11 function.

The *Hox9*, *Hox10* and *Hox11* paralog genes exhibit strong amino acid sequence homology in their homeodomains, and all nine of these genes on the HoxA, C and D clusters show strong and overlapping expression during kidney development, consistent with functional redundancy^16^. The Hox9,10,11 paralogs all evolved from a single Abdominal-B (Abd-B) type ancestral Hox gene. Further, the Hox10 and Hox11 paralog genes have been previously shown to be important in kidney development, although they have not heretofore been assayed for function with combinations of both paralogous and flanking gene mutations. Of interest, no kidney phenotype has been reported for the Hox9 genes, even after mutation of all four paralogs, likely because of the remaining functionally redundant Hox10 and Hox 11 genes^24^. Given that Hox9 paralogs are robustly expressed in conjuction with the Hox10 and Hox11 paralogs in the developing kidney, we targeted Hox9,10,11 paralogous genes to investigate possible novel core functions of Abd-B Hox genes in kidney development.

We previously made mice with mutations of the *Hoxa9*,*10*,*11* and the *Hoxd9*,*10*,*11* genes and described the resulting reproductive tract^10^ and limb^25^ malformations. We used a recombineering method that allows simultaneous frameshift mutation of multiple flanking genes^10^. This would be challenging to accomplish with CRISPR/Cas9, where concurrent mutation of nearby alleles primarily gives deletions resulting from the introduced double stranded breaks in DNA. In this report, we additionally generated mice with mutations in *Hoxc9*,*10*,*11*. These were interbred with the previous Hox mutants to generate an extensive series of multi-Hox mutant mice, and the resulting kidney phenotypes were characterized. We observed a spectrum of renal development defects, including hypoplasia, dysplasia, and renal agenesis. This work further demonstrates the functions of Hox genes in determining nephron number by regulating cap mesenchyme (CM) progenitor cell maintenance and differentiation as well as uretic bud (UB) branching^18,19^. In addition, it better defines the distinct functions of different Hox genes in regulating these processes.

Additionally, we observed that the loss of certain combinations of paralogous and flanking Hox genes produced novel phenotypes. For example, *Hoxc9*,*10*,*11*−/− *Hoxd9*,*10*,*11*−/− mutants showed highly penetrant severely cystic kidneys, while several other Hox mutation combinations produced cystic kidneys with lower penetrance. To our knowledge, this is the first connection made between Hox genes and severe cyst formation.

Disruption of Hox gene function also resulted in a striking cellular level lineage infidelity. Hox mutant nephron tubules included intermixed cells with incorrect, or in some cases ambiguous differentiation states. Flanking cells within a tubule expressed markers of distinct segment identities. Further, we observed individual tubules cells that surprisingly expressed markers associated with more than one differentiated cell type. This resembles the multi-lineage priming previously documented in earlier kidney progenitor cells^26^, only now present at a much later developmental stage than normal. These results indicate that Hox genes are required at the cellular level for the proper execution of differentiation programs, choosing and/or maintaining the correct lineage, and fully repressing genes associated with incorrect differentiation decisions.

## Results

### Generating multi-Hox mutant mice

**Figure 1:**
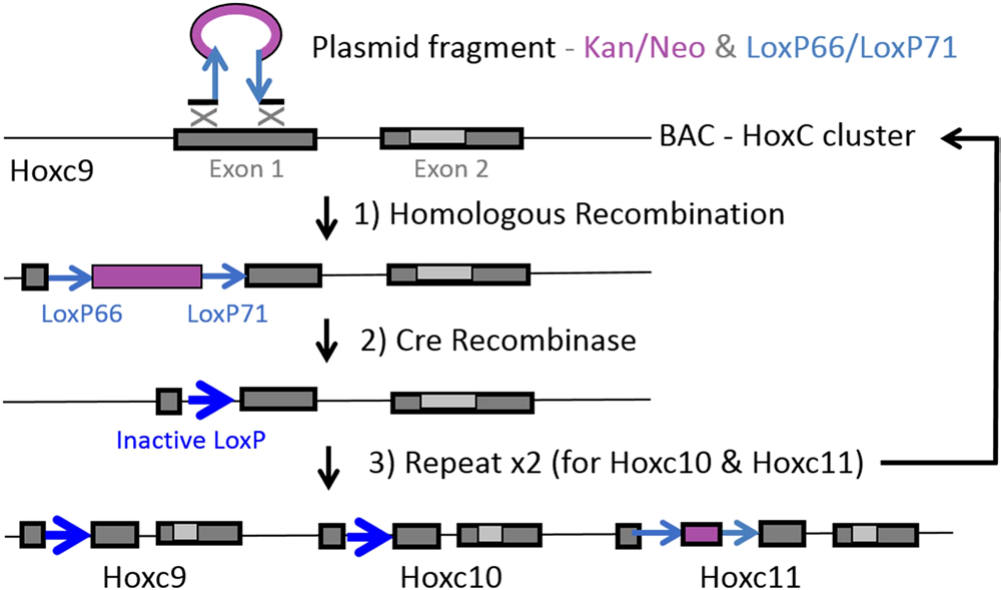
Generation of *Hoxc9*,*10*,*11* targeting construct. A modified recombineering strategy was utilized to introduce concurrent frameshift mutations in the adjacent genes, *Hoxc9*, *Hoxc10*, and *Hoxc11*. A BAC with the entire HoxC cluster was first altered at the *Hoxc9* locus through homologous recombination with a DNA segment including a Kan/Neo selectable marker flanked by the ‘once only’ LoxP sequences LoxP66 and LoxP71. Homologous recombination was driven by two DNA segments with sequence homology to coding regions of the first exon. Inducible Cre recombinase was then used to delete the Kan/Neo selectable marker, leaving behind an inactive LoxP sequence and a small deletion of coding sequence, creating a frameshift mutation. The process was then repeated for *Hoxc10* and *Hoxc11*.

We generated mice with concurrent frameshift mutations in *Hoxc9*, *Hoxc10*, and *Hoxc11* (*Hoxc9*,*10*,*11*−/−) using BAC recombineering based gene targeting technology as previously described^10^. Specifically, a BAC targeting construct was engineered to carry multiple frameshift mutations (Fig. 1, Tables S1,S2). A DNA segment with a *Kan*/*Neo* selectable marker flanked by two regions of sequence homology to the first exon of *Hoxc10* was recombined with a BAC including the entire HoxC cluster. Following identification of *E*. *coli* carrying the desired modification the marker was removed with inducible Cre recombinase. We used the ‘once only’ LoxP sequences, Lox66 and Lox71, which each carry one mutation, so that the single remaining LoxP following recombination is an inactive double mutant that did not interfere with subsequent serial modifications of *Hoxc10* and *Hoxc11*. The final BAC targeting construct was electroporated into embryonic stem cells (ESCs) which were screened by genomic DNA quantitative PCR (qPCR) to identify clones with only a single remaining wild type *Hoxc9* allele, indicating correct targeting (Fig. S1). In the resulting targeted mice the remaining *Kan*/*Neo* sequence was removed by breeding to mice with germ line Cre expression. Thus, this strategy allows us to target frameshift mutations in multiple flanking Hox genes, while leaving intergenic and intronic shared enhancers intact. We also made *Hoxa9*,*10* mutant mice (*Hoxa9*,*10*−/−), to facilitate determination of *Hoxa11* specific contributions, exactly as previously described for *Hoxa9*,*10*,*11*, only just for the *Hoxa9*,*10* genes^10^. Due to decreased fertility in multi-Hox mutant combinations, we interbred double heterozygotes (e.g. *Hoxa9*,*10*,*11*+/− *Hoxc9*,*10*,*11*+/− crossed with *Hoxa9*,*10*,*11*+/− *Hoxc9*,*10*,*11*+/−) as described in methods to generate the heterozygous/homozygous and double homozygous mutant combinations examined in this study: *Hoxa9*,*10*,*11*−/− *Hoxc9*,*10*,*11*−/−, *Hoxa9*,*10*−/−*11*+/− *Hoxc9*,*10*,*11*−/−, *Hoxa9*,*10*−/−*11*+/+ *Hoxc9*,*10*,*11*−/−, *Hoxa9*,*10*,*11*−/− *Hoxc9*,*10*,*11*+/−, *Hoxa9*,*10*,*11*+/− *Hoxc9*,*10*,*11*−/−, *Hoxc9*,*10*,*11*−/− *Hoxd9*,*10*,*11*−/−, *Hoxc9*,*10*,*11*−/− *Hoxd9*,*10*,*11*+/, *Hoxc9*,*10*,*11*+/− *Hoxd9*,*10*,*11*−/−, and triple heterozygotes *Hoxa9*,*10*,*11*+/− *Hoxc9*,*10*,*11*+/−, *Hoxd9*,*10*,*11*+/−.

### Multi-Hox mutant mice develop renal development defects (hypoplasia, dysplasia, and bilateral agenesis) as well as severely cystic kidneys

**Figure 2:**
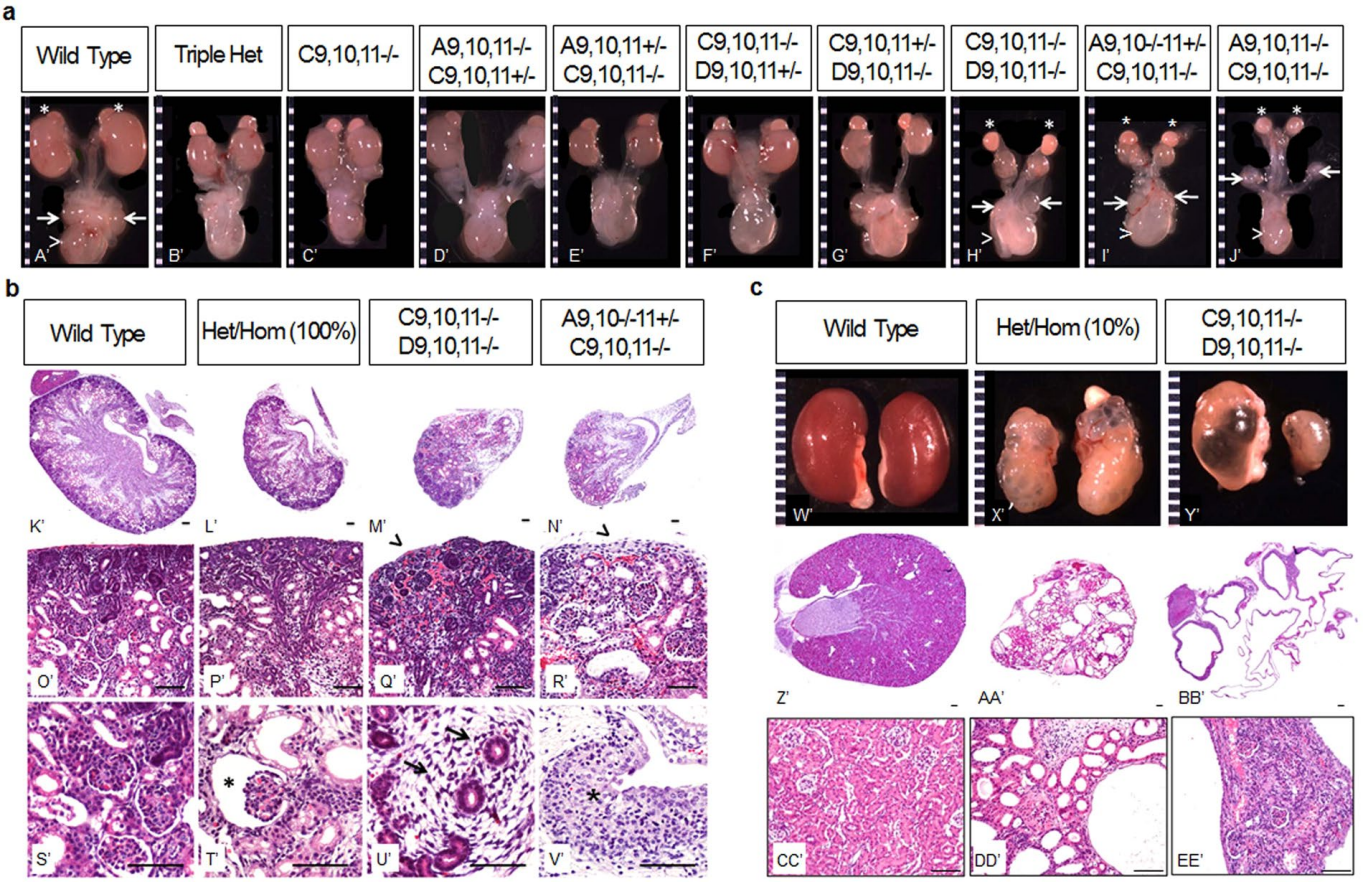
Hox mutant kidneys exhibit hypoplasia, dysplasia, and bilateral agenesis as well as severely cystic kidneys. **(a)** Gross dissections showed smaller kidneys in *Hoxc9*,*10*,*11*−/− and all heterozygous/homozygous combinations generated (*Hoxa9*,*10*,*11*+/− *Hoxc9*,*10*,*11*−/−, *Hoxa9*,*10*,*11*−/− *Hoxc9*,*10*,*11*+/−, *Hoxc9*,*10*,*11*−/− *Hoxd9*,*10*,*11*+/−, *Hoxc9*,*10*,*11*+/− *Hoxd9*,*10*,*11*−/− as well as triple heterozygotes *Hoxa9*,*10*,*11*+/− *Hoxc9*,*10*,*11*+/− *Hoxd9*,*10*,*11*+/−). *Hoxc9*,*10*,*11*−/− *Hoxd9*,*10*,*11*−/− and *Hoxa9*,*10*−/−*11*+/− *Hoxc9*,*10*,*11*−/− mutants showed very small, rudimentary kidneys and *Hoxa9*,*10*,*11*−/− *Hoxc9*,*10*,*11*−/− showed bilateral renal agenesis. N = 8 for each genotype; arrow – gonads, arrowhead – bladder, asterisk – adrenal gland. (**b)** Representative H&E images of E18.5 all heterozygous/homozygous mutants generated (genotypes listed above) showed hypoplasia (P’,T’). We observed occasional cystic tubules and dilated Bowmen’s space (T’; asterisk). *Hoxc9*,*10*,*11*−/− *Hoxd9*,*10*,*11*−/− and *Hoxa9*,*10*−/−*11*+/− *Hoxc9*,*10*,*11*−/− mutant kidneys appeared dysplastic, with lobar disorganization (M’,N’) and tubules surrounded by stroma (U’, arrows). E18.5 *Hoxa9*,*10*−/−*11*+/− *Hoxc9*,*10*,*11*−/− mutants lacked a nephrogenic zone (R’; arrowhead), whereas residual areas of nephrogenesis were present in *Hoxc9*,*10*,*11*−/− *Hoxd9*,*10*,*11*−/− mutants (Q’). *Hoxa9*,*10*−/−*11*+/− *Hoxc9*,*10*,*11*−/− mutants also demonstrated dysplastic epithelium in the renal papilla (V’; asterisk). Magnification bar = 50 μm; N = 3 for each genotype. (**c**) Gross images (W’-Y’; original magnification 6x) and H&Es (Z’-BB’ original magnification 2x; CC’-EE’ original magnification 20x) showed that 10–15% of 2 month old heterozygous/homozygous multi-Hox mutant mice (N = 40 mice total) and all surviving *Hoxc9*,*10*,*11*−/− *Hoxd9*,*10*,*11*−/− mutants (N = 3) developed cystic kidneys.

We genotyped over 900 progeny mice surviving to weaning from double heterozygous matings. No *Hoxa9*,*10*,*11*−/− *Hoxc9*,*10*,*11*−/− mice survived to weaning, and *Hoxc9*,*10*,*11*−/− *Hoxd9*,*10*,*11*−/− mice experienced approximately 90% mortality (0.6% observed vs 6.25% expected). *Hoxa9*,*10*,*11*−/− *Hoxc9*,*10*,*11*−/− mutants did not have detectable kidneys at E18.5 (Fig. 2a,J’, asterisks – adrenal glands; arrows – gonads). However, in mice with a single wild type copy of *Hoxa11* present (*Hoxa9*,*10*−/−*11*+/− *Hoxc9*,*10*,*11*−/−), renal agenesis occurred in only 16% of mutants. In the remaining 84% of mutants, we observed severely small, dysplastic kidneys with a complete lack of nephron progenitors and nephrogenic zone at E18.5 (Fig. 2b,R’). Conversely, renal agenesis was rarely observed in *Hoxc9*,*10*,*11*−/− *Hoxd9*,*10*,*11*−/− mutants (1 out of a total of 25 mutants examined), and nephrogenesis continued through E18.5 (Fig. 2b,Q’). These findings reveal divergent functions of HoxA and HoxD paralogous genes in both the maintenance of nephron progenitors as well as early formation of the metanephric kidney. Specifically, a minimal amount of *Hoxa11* is necessary for metanephric kidney development, since *Hoxa9*,*10*,*11*−/− *Hoxc9*,*10*,*11*−/− mutants fail to develop kidneys, which cannot be compensated for by the remaining *Hoxd9*,*10*,*11* paralogs. However, with the addition of a single copy of *Hoxa11* (ie: *Hoxa9*,*10*−/−*11*+/− *Hoxc9*,*10*,*11*−/−*)*, the metanephric kidney develops, though abnormally, as these mutants show early loss of nephron progenitor cells. Further, the *Hoxc9*,*10*,*11*−/− *Hoxd9*,*10*,*11*−/− mutants maintain nephron progenitors longer than *Hoxa9*,*10*−/−*11*+/− *Hoxc9*,*10*,*11*−/− mutants, giving evidence that the HoxA and HoxD cluster genes are not fully functionally equivalent.

Heterozygous/homozygous mutants showed renal hypoplasia in all the multi-Hox mutant combinations generated in this study, specifically: *Hoxa9*,*10*,*11*+/− *Hoxc9*,*10*,*11*−/−, *Hoxa9*,*10*,*11*−/− *Hoxc9*,*10*,*11*+/−, *Hoxc9*,*10*,*11*−/− *Hoxd9*,*10*,*11*+/−, *Hoxc9*,*10*,*11*+/− *Hoxd9*,*10*,*11*−/− as well as triple heterozygotes *Hoxa9*,*10*,*11*+/− *Hoxc9*,*10*,*11*+/− *Hoxd9*,*10*,*11*+/− (Fig. 2b,L’ and Supplemental Fig. S2). Interestingly, despite the different combinations of Hox gene loss, the many heterozygous/homozygous and triple heterozygous mutant phenotypes were indistinguishable at E18.5 (Fig. S3). These mutant kidneys were smaller and nephron allotment was significantly decreased (Fig. S4). Surprisingly, *Hoxc9*,*10*,*11*−/− mutants showed significantly reduced numbers of nephrons (Fig. S4), even though multiple paralogous 9,10,11 genes remained intact.

Grossly cystic kidneys were observed in all surviving *Hoxc9*,*10*,*11*−/− *Hoxd9*,*10*,*11*−/− mutants (age 3–6 weeks) (Fig. 2,Y’,BB’). Additionally, in mice uniformly sacrificed at 2 months of age, 10–15% of the *Hoxa9*,*10*,*11*−/− *Hoxc9*,*10*,*11*+/−, and *Hoxa9*,*10*,*11*+/− *Hoxc9*,*10*,*11*−/−, and triple heterozygote *Hoxa9*,*10*,*11*+/− *Hoxc9*,*10*,*11*+/− *Hoxd9*,*10*,*11*+/− mice showed grossly cystic kidneys (Fig. 2c,X’,Y’). Cysts were also observed in *Hoxc9*,*10*,*11*−/− *Hoxd9*,*10*,*11*+/− and *Hoxc9*,*10*,*11*+/− *Hoxd9*,*10*,*11*−/− mice at later adult time points (between 4 to 12 months). While small, scattered cysts were previously reported in HoxD cluster mutants^22^, this is to our knowledge the first connection made between Hox genes and severely cystic kidneys. Embyronic and adult mice were grossly examined for defects in other major organ systems, with no other anomalies identified other than the decreased fertility and kidney defects described herein.

### Ureteric bud (UB) branching and cap mesenchyme (CM) defects

**Figure 3:**
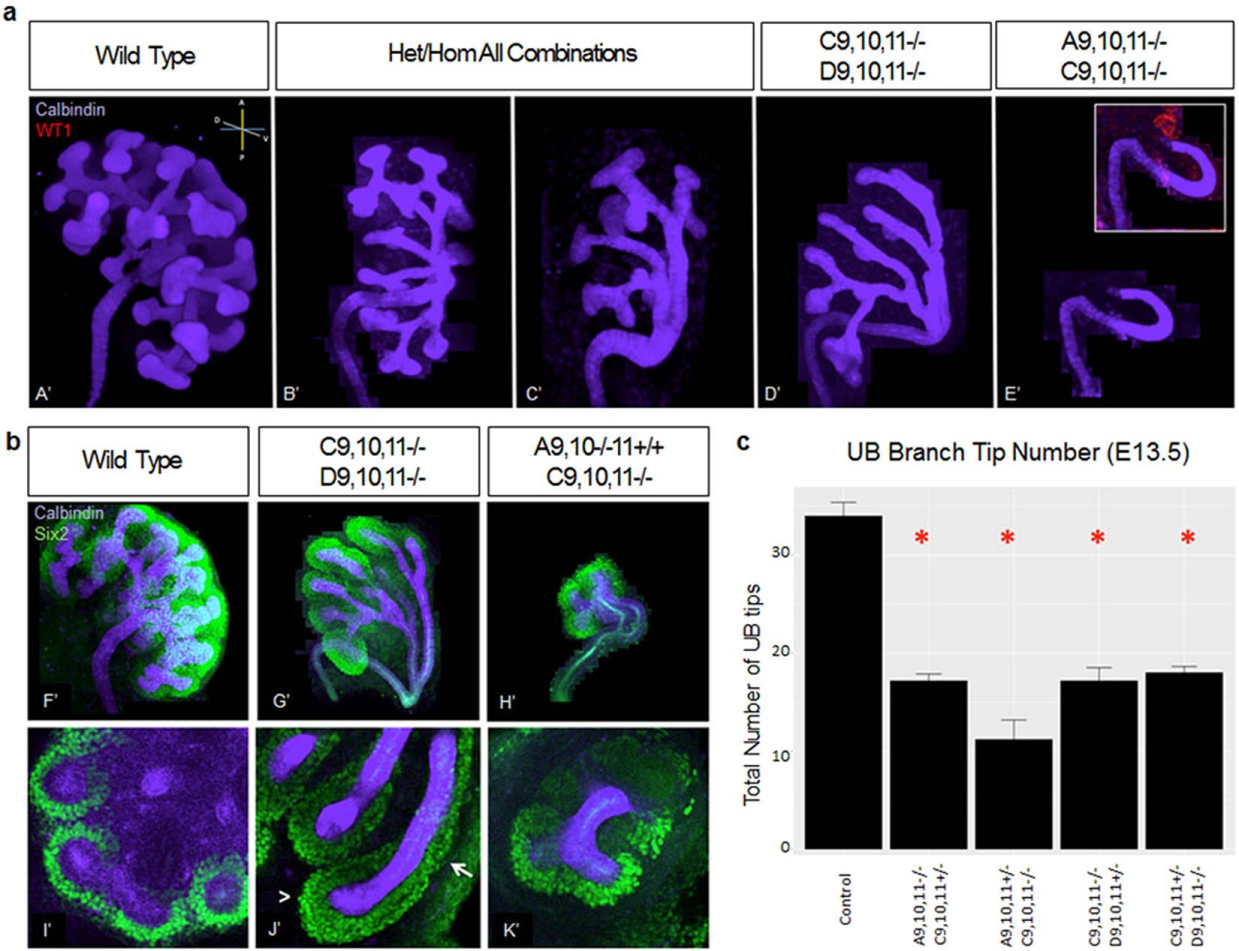
Ureteric bud (UB) and cap mesenchyme (CM) at E13.5. **(a)** All heterozygous/homozygous Hox mutant mice generated demonstrated asymmetric branching, either in the dorsal/ventral axis alone (B’) or both the anterior/posterior and dorsal/ventral axes (C’) as quantified by genotype in (Table S3). *Hoxc9*,*10*,*11*−/− *Hoxd9*,*10*,*11*−/− mutants developed elongated branches with limited secondary branching (D’, G’, J’; N = 7/8 mutants examined; with 1 mutant showing absent branching). *Hoxa9*,*10*−/−*11* +/+ *Hoxc9*,*10*,*11*−/− mutants developed a few branch tips (H’, K’; N = 3). *Hoxa9*,*10*,*11*−/− *Hoxc9*,*10*,*11*−/− mutants lacked secondary branching (E’; N = 3) with a single glomerulus (insert; Wt1 – red). (**b)** *Hoxc9*,*10*,*11*−/− *Hoxd9*,*10*,*11*−/− kidneys showed increased thickness of CM (J’, arrowhead; Six2 – green) surrounding the reduced number of UB tips (calbindin – purple). CM showed abnormal extension down the length of the UB branch/stalk (J’, arrow). *Hoxa9*,*10*−/−*11* +/+ *Hoxc9*,*10*,*11*−/− kidneys were severely affected in terms of both UB branching and CM mass (H’,K’). (**c**) Decreased UB branch tips were observed in all heterozygous/homozygous Hox combinations (N = 3 per genotype, asterisk indicates p < 0.05 from control).

Heterozygous/homozygous mutants showed dramatically reduced, asymmetric branching (Fig. 3a), which was quantified by genotype (Table S3). Some mutants demonstrated dorsal/ventral axis asymmetry (Fig. 3a,B’) with branching primarily along the dorsal axis, while other Hox mutant combinations were more affected, with isolated branching only in the dorsal and anterior axes (Fig. 3a,C’). Interestingly, *Hoxc9*,*10*,*11*−/− *Hoxd9*,*10*,*11*−/− mutants consistently developed elongated UB stalks extending anteriorly (Fig. 3a,b; D’,G’). There was no apparent deficit in early CM formation in *Hoxc9*,*10*,*11*−/− *Hoxd9*,*10*,*11*−/− mutants, with abundant CM surrounding the reduced number of UB branches present at E13.5 (Fig. 3b,G’,J’). Nephrogenesis, however, appeared impaired, with the CM extending along almost the entire length of some elongated UB branches (Fig. 3b,J’). At E13.5, *Hoxa9*,*10*−/− *11*+/+ *Hoxc9*,*10*,*11*−/− mutants showed an even more severe UB defect, with very few branches despite intact *Hoxa11* and *Hoxd11* function (Fig. 3b,H’,K’). Interestingly, *Hoxa9*,*10*,*11*−/− *Hoxc9*,*10*,*11*−/− mutants, with complete loss of *Hoxa11* function, developed a single glomerulus (marked by Wt1 staining) at the end of the unbranched UB outgrowth (Fig. 3a,E’ insert). All combinations of heterozygous/homozygous mutants appeared to have grossly normal CM at E13.5, E15.5, and E18.5 (data not shown).

### Severe disruption of Hox9,10,11 function results in lineage infidelity

In *Drosophila*, Hox genes are known to drive segment identity determination, with mutations often resulting in dramatic homeotic transformations. In mammals, single Hox mutations generally give more subtle phenotypes, likely due to Hox functional redundancies. The mice in this study in some cases carried large numbers of both flanking and paralogous Hox gene mutations, leading us to suspect that they might show abnormalities in nephron segment identity determination.

**Figure 4:**
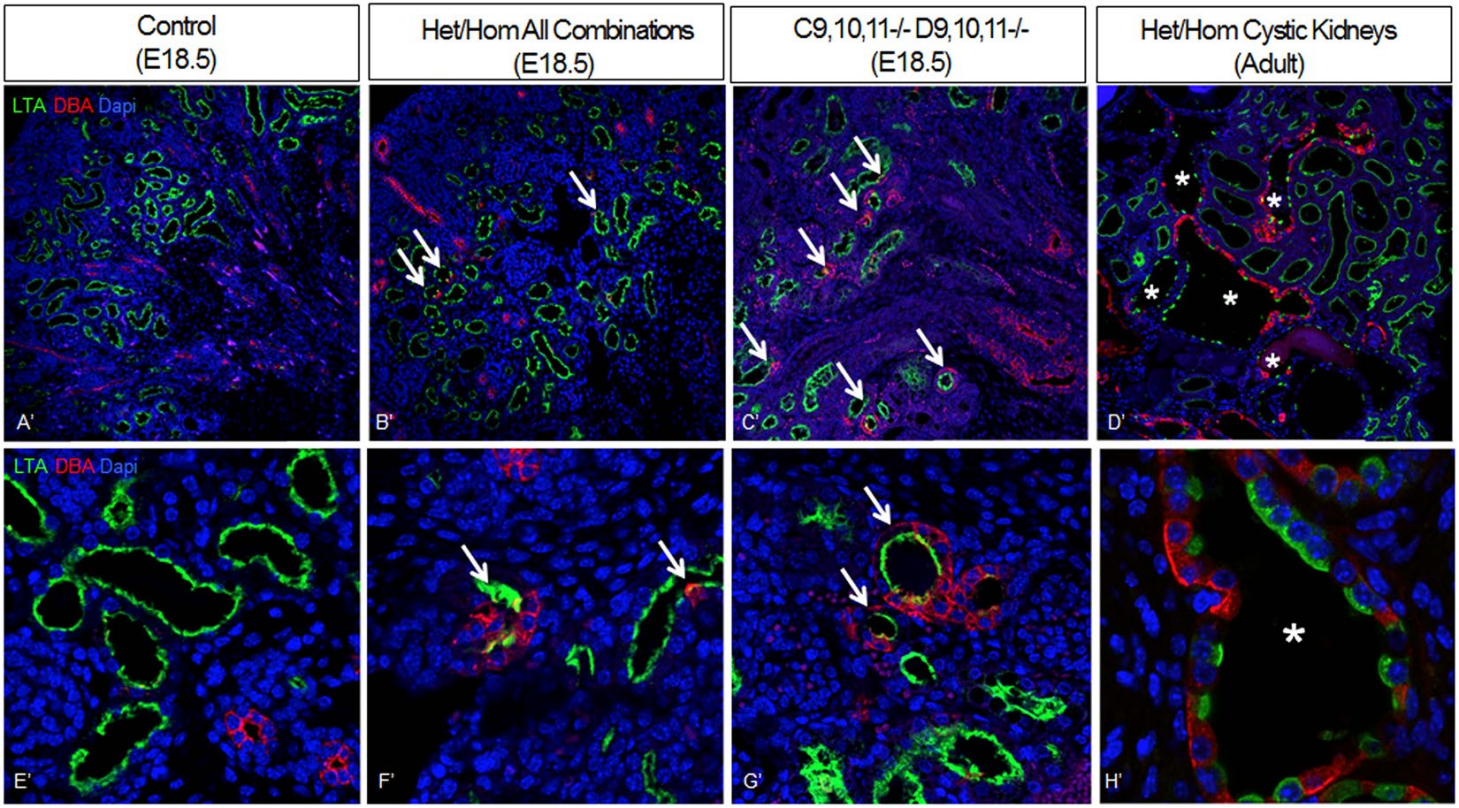
Hox mutant kidney tubules develop ambiguous nephron segmentation. Intermixed DBA (collecting duct) and LTA (proximal tubule) positive cells were present in E18.5 all heterozygous/homozygous mutants generated (*Hoxa9*,*10*,*11*−/− *Hoxc9*,*10*,*11*+/−, *Hoxa9*,*10*,*11*+/− *Hoxc9*,*10*,*11*−/−, *Hoxc9*,*10*,*11*−/− *Hoxd9*,*10*,*11*+/−, *Hoxc9*,*10*,*11*+/− *Hoxd9*,*10*,*11*−/−) (B’,F’; arrows) but were more frequent in *Hoxc9*,*10*,*11*−/− *Hoxd9*,*10*,*11*−/− embryonic kidneys (C’,G’; arrows) and *Hoxa9*,*10*−/−*11*+/− *Hoxc9*,*10*,*11*−/− (data not shown). Adult Hox mutants that develop cysts showed numberous tubules with intermixed DBA and LTA positive cells (H’,L’); (asterisk).

We first used lectin staining to examine nephron segmentation and cystic tubular segments in the Hox mutant kidneys. Surprisingly, in E18.5 *Hoxc9*,*10*,*11*−/− *Hoxd9*,*10*,*11*−/− mutants there were many tubules with cells that dual labeled with LTA, a proximal tubule marker lectin, as well as DBA, a collecting duct marker (Fig. 4,C’,G’). This was also observed in *Hoxa9*,*10*−/−*11*+/− *Hoxc9*,*10*,*11*−/− mutants (data not shown) and at a lower frequency, in all the heterozygous/homozygous combinations generated, with fewer mutant Hox genes (Fig. 4,B’,F’). In some cases, cells showed a mixed identity, expressing both LTA and DBA, while in other cases flanking cells in a tubule showed different identities. This lineage infidelity persisted in adults, but only in the cystic kidneys (Fig. 4,D’,H’).

To further investigate the ambiguities in nephron segmentation the multi-Hox mutant kidneys were examined with a thorough battery of nephron segment specific markers, including *Lotus tetragonolobus* agglutinin (LTA), HNF4a, and Villin specific to the proximal tubule^27–29^, SLC12A1 which is expressed in the ascending loop of Henle^30,31^, *Dolichos biflorus* agglutinin (DBA)^27,32^ and Cytokeratin 8 (CK)^33,34^ which are specific for the collecting duct, AQP2 which is expressed in the connecting tubule and collecting duct^35^, and SLC8A1 which is expressed in both the distal tubule and connecting tubule^36^.

**Figure 5:**
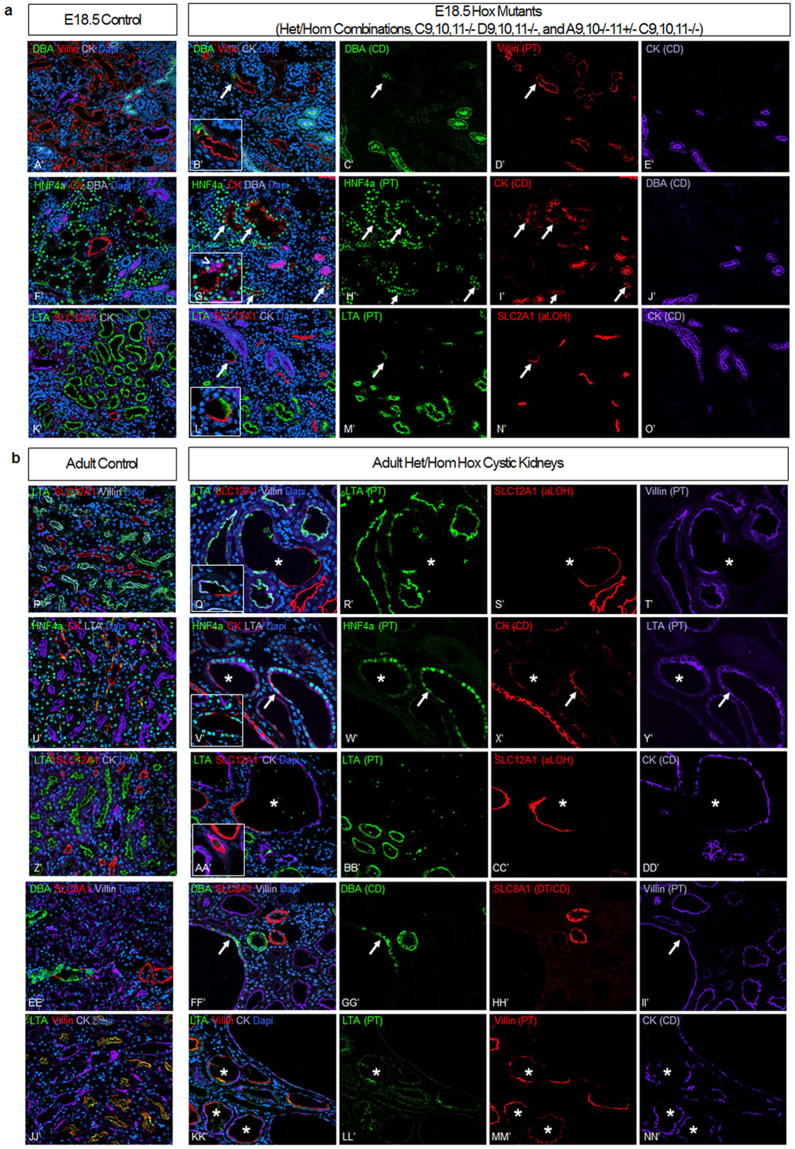
Multi-Hox mutant kidney tubules have intermixed cells with distinct nephron segment identities as well as single cells that express markers of more than one segment. (**a**) At E18.5, we observed tubules containing DBA (collecting duct) cells interspersed among Villin (proximal tubule) positive cells (B’). Multi-Hox mutants also showed single cells that triple labeled with HNF4a (proximal tubule) as well as DBA and CK (collecting duct) (G’, insert, arrowhead). LTA (proximal tubule) and SLC12A1 (ascending loop of Henle) positive cells were present in a single tubule (L’). (**b**) Adult cystic kidneys showed cells with LTA and Villin (proximal tubule) adjacent to SLC12A1 (ascending loop of Henle) cells (Q’). Single cells also co-expressed HNF4a (proximal tubule) with CK (collecting duct) (V’), SLC12A1 (ascending loop of Henle) with CK (collecting duct marker) (AA’), and Villin (proximal tubule) with DBA (collecting duct marker) (FF’). Cysts also showed regional expression of proximal tubule markers (Villin and/or LTA) adjacent to CK cells (collecting duct) (KK’). N = 3 per immunoassay.

In control kidneys, the cells of a tubule uniformly labeled with a single segment identity marke as expected (Fig. 5a,b). In Hox mutants, however, we surprisingly observed single cells and clusters of cells within tubules that showed unexpected expression patterns of segment specific markers. We anticipated that an entire nephron segment might be altered in identity, or absent, but instead we observed lineage infidelity at the cellular level. These findings were not only validated using multiple markers in the adult multi-Hox mutant cystic kidneys, but were also present in embryonic kidneys in both the most severely affected genotypes (*Hoxc9*,*10*,*11*−/− *Hoxd9*,*10*,*11*−/− and *Hoxa9*,*10*−/−*11*+/− *Hoxc9*,*10*,*11*−/− mutants) as well as the less affected heterozygous/homozygous combinations. For example, we observed that some cells within a tubule expressing Villin, a proximal tubule marker, were positive for DBA, a collecting duct marker (Fig. 5a,B’). Similarly, cells expressing HNF4a (proximal tubule) also expressed CK (collecting duct) (Fig. 5a,G’). A few single cells triple labeled with HNF4a as well as DBA and CK (G’, insert, arrowhead), suggesting strong signatures of both a collecting duct cell (with the expression of two markers) as well as a proximal tubule (with expression of the nuclear transcription factor HNF4a). Additionally, cells within a tubule positive for the proximal tubule marker LTA showed co-expression of the ascending loop of Henle marker SLC12A1 (Fig. 5a,L’).

Similarly, in adult cystic kidneys, we observed unexpected expression patterns of segment specific markers. Tubules showed cells with LTA and Villin (proximal tubule markers) adjacent to cells expressing SLC12A1 (ascending loop of Henle) (Fig. 5b,Q’). Single cells co-expressed HNF4a (proximal tubule transcription factor) with CK (collecting duct marker) (Fig. 5b,V’), as well as SLC12A1 (ascending loop of Henle) with CK (collecting duct marker) (Fig. 5b,AA’), and Villin (proximal tubule) with DBA (collecting duct marker) (Fig. 5b,FF’). We also observed tubules with regional expression of proximal tubule markers (Villin and/or LTA) adjacent to CK cells (collecting duct) (Fig. 5b,KK’). Additionally, we observed SLC8A1 (distal/connecting tubule) and AQP2 (connecting tubule/collecting duct) positive cells with interspersed LTA positive cells; however, the LTA cells did not co-express two other proximal tubule markers HNF4a or Villin, suggesting in this case a partial shift in identity (data not shown). The observed frequent clustering of cells with mixed identity suggests a clonal origin. The combination of proximal tubule and collecting duct identity was particularly unanticipated since the proximal tubule is derived from the CM while the collecting duct originates from the UB lineage.

**Figure 6:**
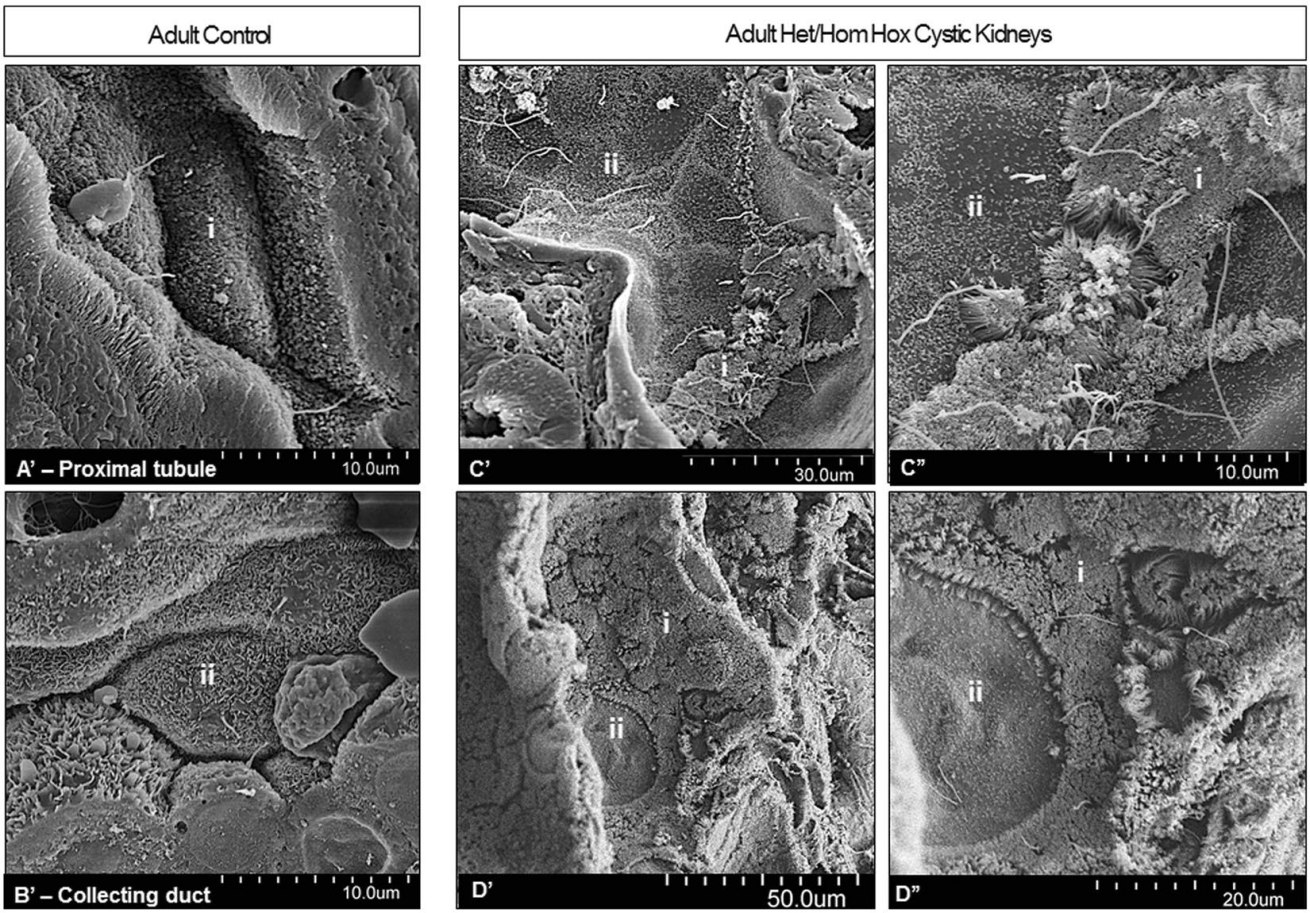
Scanning electron microscopy confirmed abnormal cell morphologies in adult multi-Hox mutant cystic kidneys. Mutant tubules showed regions of tightly packed microvilli consistent with proximal tubule brush border (i) interspersed among distal tubule/collecting duct cells^37^ (ii) with sparse microvilli (C’; higher magnification in C”). Conversely, cystic tubules consisting of primarily proximal tubule cells (i) contained interspersed cells with short, sparse microvilli^37^ (ii) consistent with distal tubule/collecting duct cells^28,37^ (D’; higher magnification in D”), not observed in control kidney proximal tubules (A’) or collecting ducts (B’). N = 3 controls and 4 mutants.

We also used scanning electron microscopy (SEM) to further confirm the unexpected intermixing of cell type morphologies. In all the Hox mutant cystic kidneys examined (*Hoxa9*,*10*,*11*−/− *Hoxc*,*10*,*11*+/−, *Hoxa9*,*10*,*11*+/− *Hoxc*,*10*,*11*−/−, *Hoxc9*,*10*,*11*−/− *Hoxd*,*10*,*11*+/−, and *Hoxa9*,*10*,*11*+/− *Hoxc*,*10*,*11*+/− *Hoxd9*,*10*,*11*+/−), we consistently observed cells with brush-border like microvilli characteristic of proximal tubules (i) interspersed among distal tubule/collecting duct cells with distinctive short, sparse^37^ microvilli (Fig. 6,C’, higher magnification in C”). We also observed the reverse, with distal tubule/collecting duct morphology cells intermixed with proximal tubule type cells (Fig. 6,D’, higher magnification in D”). Such patchworks of intermingled cell types were not observed in wild type kidneys (Fig. 6, proximal tubule in A’ and collecting duct in B’).

### RNA-seq defines global gene expression changes in Hox mutant kidneys

To provide a more global view of the altered gene expression state of the Hox mutant kidneys, we performed RNA-seq on E18.5 kidneys of wild type and *Hoxa9*,*10*−/−*11*+/− *Hoxc9*,*10*,*11*−/− and *Hoxa9*,*10*−/−*11*+/+ *Hoxc9*,*10*,*11*−/− mutants (Table S4). Down-regulated genes included *Cited1* and *Six2*, markers of the CM, likely reflecting the reduced CM representation in these mutants. Up-regulated genes included *Postn*, *Lum*, *Dcn*, *Lox*, *Eln*, and multiple collagens, all associated with extracellular matrix. The elevated levels of LOX, DCN, CollVI, and CLDN11 in E18.5 *Hoxa9*,*10*−/−*11*+/− *Hoxc9*,*10*,*11*−/− mutants were validated using immunofluorescence^38^ (Fig. S5).

**Figure 7:**
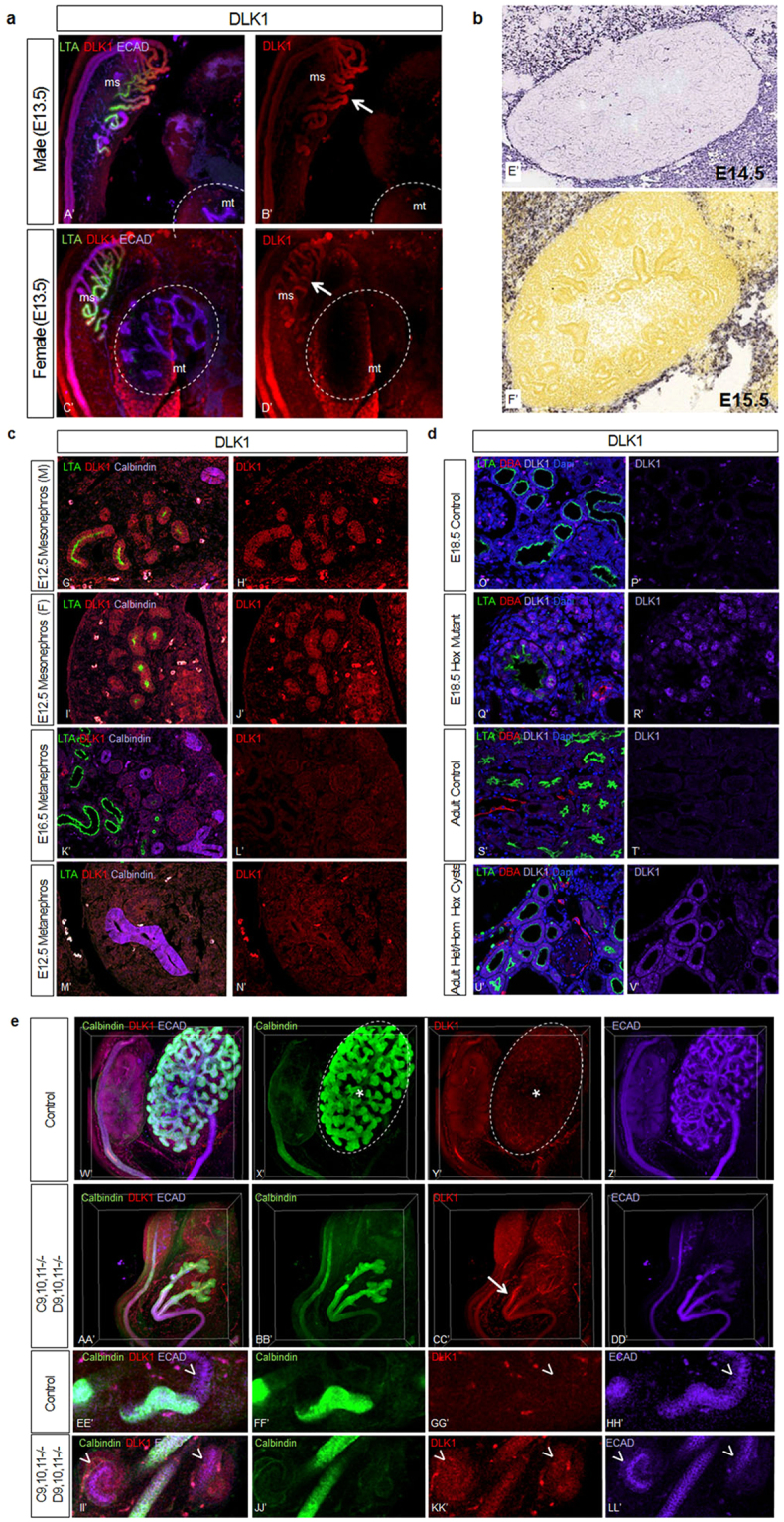
Dlk1 expression is upregulated in Hox mutant kidneys. Dlk1 expression is very low in the wild type metanephros at E12.5 (N’), E13.5 (B’,D’), E14.5 (E’, image from Eurexpress.org), E15.5 (F’, image from Allen Brain Atlas, brain-map.org), E16.5 (L’), E18.5 (P’) and adult (T’). In contrast, the wild type mesonephros shows DLK1 expression at E12.5 (G’–J’). *Hoxa9*,*10*−/−*11*+/− *HoxC*−/− mutant kidneys show DLK1 expression in multiple cell types, including proximal tubules, as well as interstitial cells (Q’,R’), and DLK1 is upregulated in Hox cystic kidneys as well (U’,V’). *Hoxc9*,*10*,*11*−/− *Hoxd9*,*10*,*11*−/− show increased DLK1 expression along the ureteric stalks (CC’, arrow) and in developing nephrons (KK’, arrowheads) in E13.5 whole mount IF. N = 3 per immunoassay.

Of particular interest, one of the most strongly upregulated genes in mutants was *Dlk1* (9.5 FC). This gene encodes a noncanonical Notch ligand that functions as an inhibitor of Notch signaling *in vitro* and is an important regulator of the differentiation of adipocytes and several other cell types^39–42^. Notably, Notch signaling is critically important for nephrogenesis, required for the formation of all nephron segments and not just proximal regions^43^. The expression of *Dlk1* is very low in the wild type metanephros at E13.5, E14.5, E15.5 and E18.5, as measured by *in situ* hybridization and IF (Fig. 7a–d). We confirmed the elevated expression of DLK1 in the E18.5 *Hoxa9*,*10*−/−*11*+/− *Hoxc9*,*10*,*11*−/− mutants by IF, in both proximal tubule (LTA positive) and interstitial cell types at E18.5 and in adult Hox mutant cystic kidney tubules (Fig. 7d,R’,V’). We also observed up-regulation of DLK1 in *Hoxc9*,*10*,*11*−/− *Hoxd9*,*10*,*11*−/− mutants, both in the ureteric stalks as well as developing nephrons at E13.5 (Fig. 7e,CC’). Of interest, in wild type controls we observed Dlk1 expression in the more anterior, primitive mesonephros, but not in the metanephros, which gives rise to the adult kidney (Fig. 7c). These results identify *Dlk1* as a novel marker that distinguishes meso and metanephros, and give evidence for Hox regulation of the Notch signaling pathway in the developing kidney.

## Discussion

Functional analysis of mammalian Hox genes is challenging because of their extensive redundancies. It has been observed that mutation of a single Hox gene often results in a mild phenotype, while removal of combinations of related Hox genes can give a more dramatic phentoypes. In this report we pursue this general principle by introducing frameshift mutations into an extensive series of Abd-B type Hox genes, thereby uncovering novel Hox functions in kidney development.

It is interesting to note that Hox genes have been proposed to have more evolutionarily primitive organogenesis functions that are distinct from segment identity determination^44^. Hox genes can promote cell proliferation^45,46^ and regulate cell death^47^. In some cases it has been shown that Hox gene mutations can result in defective cell differentiation that is unrelated to segment identity^48^. Of particular note, in the Drosophila developing larval fat body it has been shown that Hox genes have a shared function in repressing autophagy^49^. Hox genes show extensive overlapping expression in this structure, with common anterior limits, suggesting a function unrelated to segmentation. Repression of all Hox gene expression is required for the onset of normal autophagy during development, while continued expression of any Hox gene can block this process^49^.

In the developing mouse kidney, there is also extensive overlap of Hox gene expression, with no apparent Hox expression codes that might drive compartment/segment specific differentiation^16,50^ (Magella and Potter, unpublished observations). In this report, in order to uncover possible Hox gene shared functions previously concealed by redundancies we generated and analysed mice with multiple combinations of frameshift mutations in nine very closely related Abd-B type Hox genes. The use of frameshift mutations preserved shared intergenic enhancers driving expression of the remaining Hox genes. The *Hoxa9*,*10*,*11*, *Hoxc9*,*10*,*11* and *Hoxd9*,*10*,*11* genes all show strong expression in very similar domains during kidney development. The Hox9 mutants have no reported kidney defects, even when all four paralogs are mutated, likely a result of remaining Hox genes with overlapping function^24^, while Hox10 mutant kidneys show stromal defects when all three paralogs are mutated^24^, and Hox11 mutant kidneys have cap mesenchyme/UB branching defects that result in reduced nephron number, with renal agenesis when all paralogs are mutated^16,17,19^. Hox11 proteins complex with PAX2/EYA1 to regulate *Six2* and *Gdnf* expression^51^. *Six2* is required for CM progenitor cell maintenance^52,53^, and GDNF drives UB branching morphogenesis^54^. In summary, while many paralog specific roles for Hox genes have been defined, the premise of the current study is that by mutating new combinations of the Hox9,10,11 genes, including Abd-B type genes from multiple paralog groups, it might be possible to reveal new shared/core Hox functions previously concealed by redundancy.

Indeed, the resulting Hox9,10,11 mutant phenotypes included a number of unexpected features. For example, homozygous mutation of only the *Hoxc9*,*10*,*11* genes gave a smaller kidney with a significantly reduced nephron count, despite the remaining presence of many Hox9,10,11 paralogous genes. This gives further evidence for overlapping function of flanking Hox genes, providing additional justification for studies including mutations in both adjoining and paralogous Hox genes. Also of interest, *Hoxa9*,*10*−/−*11*+/− *Hoxc9*,*10*,*11*−/− mutants showed earlier depletion of CM than *Hoxc9*,*10*,*11*−/− *Hoxd9*,*10*,*11*−/− mutants, indicating that these Abd-B HoxA genes are more important for CM maintenance than the HoxD genes. Further, we observed a dramatic upregulation of *Dlk1* expression in the Hox9,10,11 mutant kidneys, giving evidence for a Hox/Notch link during kidney development. In addition *Hoxc9*,*10*,*11*−/− *Hoxd9*,*10*,*11*−/− mice, with homozygous mutation of six Hox genes, showed highly penetrant severe cyst formation, while mice with fewer Hox genes mutations also showed cysts but with lower penetrance.

While this study identifies novel Hox gene functions through the mutation of many different combinations of Abd-B Hox genes, there is clearly much work left to do in terms of defining the contributions of specific Hox genes. For example, to better characterize Hox9 functions it would be useful to make mice with mutations in combinations of Hox9 and Hox10 genes, as well as mice with mutations in Hox9 and Hox11 genes, and also mice with concurrent mutations in Hox10 and Hox11 genes. A comparison of phenotypes showing a synergistic severity when Hox9 mutations were added to Hox10 or Hox11 mutations would indicate Hox9 functional overlap. Indeed, the results presented in this study do not prove any kidney development function for the Hox9 genes, although their sequence homology and overlapping expression patterns with the Hox10,11 genes are strongly suggestive.

**Figure 8:**
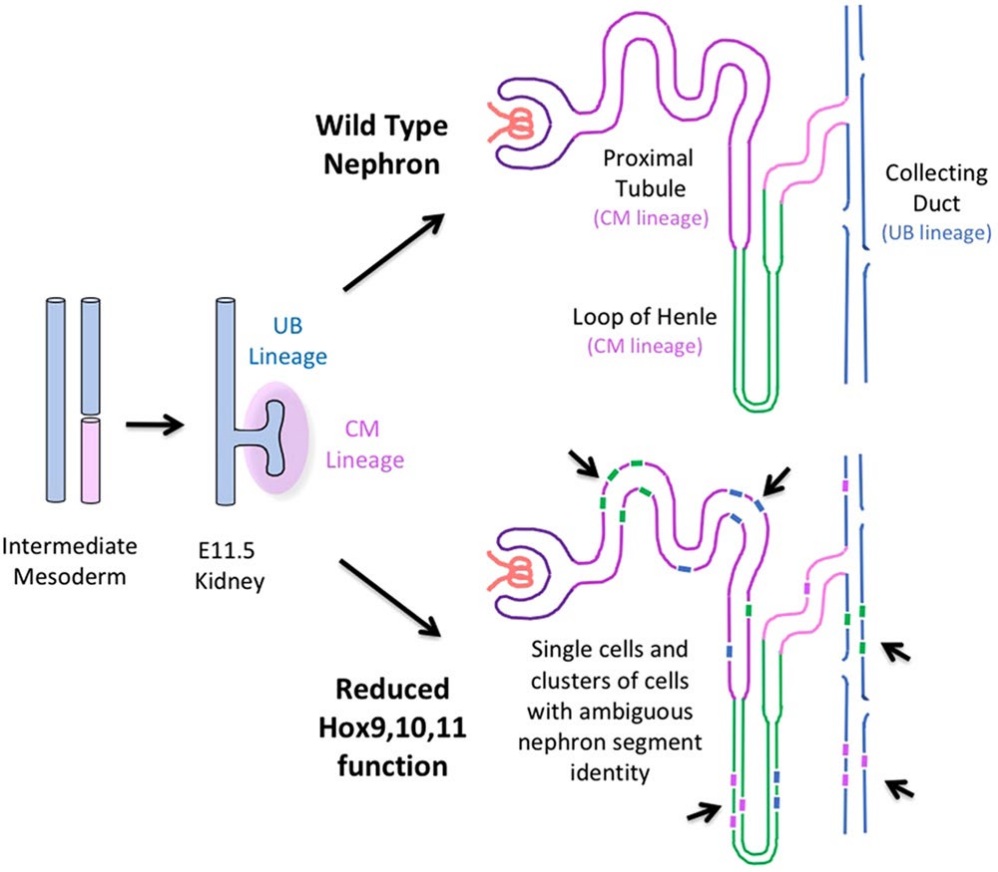
Severe disruption of Hox9,10,11 function results in cellular level lineage infidelity. *Hoxc9*,*10*,*11*−/− *Hoxd9*,*10*,*11*−/− (as well as other multi-Hox mutant combinations of *Hoxa9*,*10*,*11 Hoxc9*,*10*,*11* and *Hoxc9*,*10*,*11 Hoxd9*,*10*,*11)* renal tubules consisted of intermixed cells with proximal tubule, loop of Henle, and collecting duct identities, with some single cells expressing markers associated with more than one nephron segment.

The most surprising result of this study was the observed striking single cell level lineage infidelity. Within kidney tubules, there were cells expressing segment identity markers that did not match their neighbors. Moreover, some cells, or clusters of cells, showed ambiguous identities, simultaneously expressing genes associated with multiple segments. These results define a new role for Hox genes, in driving and/or maintaining correct differentiation decision processes at the single cell level. This is the first report, to our knowledge, of cellular level lineage infidelity in a Hox mutant. Hox genes have been thought to act at the level of the segment, and not the single cell. This single cell level disorganized differentiation was confirmed using an extensive battery of segment specific identity markers, as well as scanning electron microscopy, which demonstrated an intermixing of cells with distinct segment specific morphologies within Hox mutant tubules, as depicted in Fig. 8.

The observed variability in selected differentiation programs between neighboring cells suggests some stochastic process at work. Perhaps the simultaneous mutation of multiple Hox genes causes a severe disruption of the Hox code, resulting in an unstable and disordered state, allowing cells to move down more than one developmental pathway, or in some cases giving rise to single cells with features of more than one nephron segment cell type. It is also interesting to note that inhibition of Rho-Kinase can disrupt normal cell migration, but not differentiation, resulting in some mixing of distinct nephron segment cell types^55^. The Hox mutants in this report, however, include single cells with mixed lineage, as well as flanking cells with identities from non-contiguous nephron segments, arguing against simple mixing.

Alternatively, the results could be interpreted in terms of a severe reduction of the Hox dose, instead of a disruption of the Hox code. We have previously shown that during kidney development nephron progenitors exhibit multi-lineage priming, with single cells showing stochastic expression of genes associated with multiple possible future differentiation directions^26^. As development proceeds and a specific lineage is selected this uncertain state resolves, with the genes of inappropriate differentiation programs more firmly repressed, and the genes of the chosen lineage more fully activated. In mice with many mutant Hox genes this lineage decision-making process may be disrupted, resulting in either incorrect cell identities that do not match their neighbors, or in ambiguous differentiation states at much later stages in development than normally observed. It is also possible that the problem is primarily one of maintenance of the proper differentiated state. Of interest, *Hoxa9*,*10*,*11*/*Hoxd9*,*10*,*11* mutant kidneys, not included in this report, show a similar lineage infidelity phenotype (Magella *et al*. in press).

While cystic epithelium has been shown to de-differentiate, ambiguous segmentation has not been described to our knowledge as a phenomenon of cystogenesis in other cystic kidney disease models^56–58^. However, one report describes dual LTA and DBA positive cells in early postnatal renal cysts that disappear in older mice^59^. In contrast, the multi-Hox mutant kidneys show severe lineage infidelity at embryonic timepoints, before the onset of cysts, that persist in cystic tubules of adults. It is possible therefore that the cells of cysts are sensitized to differentiation ambiguity, explaining the dramatic mixing of cell types observed in the adult Hox mutants, and it is interesting to consider whether or not these defects in differentiation may relate to cyst formation.

We observed a Hox mutant dose response relationship to lineage infidelity, with more Hox gene mutations resulting in more lineage infidelity. For example, the E18.5 *Hoxc9*,*10*,*11*−/− *Hoxd9*,*10*,*11*−/− mutants showed frequent lineage infidelity, with most proximal tubules (as defined by predominant LTA staining), including some surprising cells with collecting duct (DBA staining) character; however, when fewer total Hox alleles were mutated (as in heterozygous/homozygous multi-Hox mutants), there were fewer cases of lineage infidelity (Fig. 4). Taken together, this suggests that Hox genes, in addition to their previously defined roles, could function in widespread epigenetic regulation, contributing to the conversion of poised genes, with both active and repressive histone marks, to the fully repressed or fully active state. These results better define Hox gene contributions that could help direct iPSC differentiation to kidney tissue.

In conclusion, the extensive removal of both flanking and paralogous Hox genes serves to better reveal their multifunctional character. Cells with extreme Abd-B Hox9,10,11 function removal showed a stochastic lineage infidelity, with adjacent cells showing diverse, or ambiguous, differentiation states, extending the known roles of Hox gene function in organogenesis.

## Methods

### Recombineering

A modified bacterial artificial chromosomes (BACs) recombineering strategy was used to introduce simultaneous frameshift mutations in flanking Hox genes, as previously described^10,60^. To make the *Hoxc9*,*10*,*11* mutants (Fig. 1), we made a BAC targeting construct over 100 Kb in length using clone BMQ257-D8 (HoxC cluster) from CHORI BACPAC Resources (http://bacpac.chori.org/home.htm). Plasmid constructs for recombineering of BACs were made by subcloning PCR-amplified DNA into a modified pl451^10^. with Kan/Neo flanked by ‘once only’ Lox66 and Lox71. Sequences were engineered to introduce a small deletion/frameshift mutation within each first exon. Recombineering modification of BACs was carried out as previously described^60^. Primers for PCR amplification of homology regions were designed for *Hoxc9*, *Hoxc10*, and *Hoxc11* (Table S2). The modified BACs were electroporated into SE2 ES cells (made in Potter lab), as previously described^10^. Mice were maintained on a mixed 129/CD1 background (CD1 from Charles River). The genotypes of adult and embryonic mice were determined by PCR using Direct-PCR lysis reagent (Viagen Biotech) and proteinase K (Sigma-Aldrich). All experiments were carried out with humane protocols (protocol number 2015–0065) approved by the Cincinnati Children’s Institutional Animal Care and Use Committee (IACUC) in accordance with applicable federal, state and institutional regulations as well as meeting AAALAC accreditation standards.

Heterozygous Hox mutants (such as *Hoxa9*,*10*,*11*+/−) are hypofertile with smaller litter sizes. Homozygous Hox mutants (such as *Hoxa9*,*10*,*11*−/−) are more severely affected, with some combinations showing infertility. Therefore, to generate double homozygous mutants, we interbred double heterozygotes (ie: *Hoxa9*,*10*,*11*+/− *Hoxc9*,*10*,*11*+/− females crossed with *Hoxa9*,*10*,*11*+/−, *Hoxc9*,*10*,*11*+/− males). The following crosses were used to generate the muli-Hox mutants described: *Hoxa9*,*10*,*11*+/− *Hoxc9*,*10*,*11*+/− x *Hoxa9*,*10*,*11*+/− *Hoxc9*,*10*,*11*+/−, *Hoxa9*,*10*+/−*11*+/+ *Hoxc9*,*10*,*11*+/− x *Hoxa9*,*10*,*11*+/− *Hoxc9*,*10*,*11*+/−, *Hoxc9*,*10*,*11*+/− *Hoxd9*,*10*,*11*+/− x *Hoxc9*,*10*,*11*+/− *Hoxd9*,*10*,*11*+/−, and *Hoxa9*,*10*,*11*+/− *Hoxc9*,*10*,*11*+/− x *Hoxc9*,*10*,*11*+/− *Hoxd9*,*10*,*11*+/−.

### Gross and histologic analyses

For embryos, noon of day of vaginal plug was designated E0.5. Kidney size was determined using ImageJ software by measuring the longest longitudinal length (calibrating each image to mm ruler) and averaging both kidneys for each embryo to confirm decreased size observed in gross images (data not included). Littermate wild type and single heterozygotes were used as controls. Embryonic tissue was fixed overnight in 4% PFA, washed with PBS, and stored in methanol at −20 °C. Two month old mice were sacrificed, left kidneys were perfused fixed with 2.5% glutaraldehyde/2% PFA and used for histology or electron microscopy; non-perfused right kidneys were used for histology and immunofluoresence. Tissue for histology was fixed overnight in 4% PFA followed by alcohol dehydration and paraffin embedding. Kidneys sections (3 µm) were stained with H&E according to standard protocols. Minimum 3 kidneys from each genotype were examined. Nephron numbers were quantified in 2 week old mice and littermate controls. Each kidney was processed separately using acid maceration technique^61^.

### Scanning electron microscopy (SEM)

Adult mouse kidney was perfused fixed with 2.5% glutaraldehyde/2% PFA, dehydrated in ethanol, and fractured by dropping the tissue into liquid nitrogen and breaking with a razor blade. Fractured pieces were hydrated through graded alcohol washes, washed in sodium cacodylate buffer, and fixed in 1% osmium tetroxide for 1 hour. Samples were then washed in buffer, dehydrated, treated with a critical point dryer, sputter coated with gold palladium, and visualized (Hitachi SU8010 Field Emission scanning electron microscope).

### Immunofluorescence

Immunofluorescent/lectin staining of wild type and mutant paraffin sections was carried out on the same slide, and visualization carried out with the same microscope/photography settings (NikonA1 inverted confocal microscope). Whole mount E13.5 kidneys/gonads were fixed overnight in 4% PFA, washed with PBS, and stored at −20 degrees in methanol. Staining was performed using PBST washes, incubations for 48 hours in primary followed by secondary antibody, and samples were imaged in RIMS (refractive index matching solution prepared from histodenzTM (Sigma-Aldrich) in 0.02 M phosphate buffer with 0.01% sodium azide, pH to 7.5) in a chamber slide (Ibidi). Antibodies and lectins are listed in supplementary material (Table S5).

### RNA Sequencing

RNA was isolated from E18.5 whole kidneys (N = 3 controls and 3 mutants) using RNeasy Miniprep kit (Qiagen). Illumina TruSeq RNA-Seq was performed, paired end 75 bp, with a minimum of 20 million reads per sample. Data were analyzed using Strand NGS software. Bam files were generated using mouse build mm10. Data was filtered on read quality metrics, including removal of reads aligning to more than one position in the genome, requiring >10 NRPKM in 3 of 12 samples, Audic Claverie analysis, P < 0.05, FC > 2.

### Data Availability

Data was submitted to the GEO database (GSE90816).

### Statistical analysis

Results from E18.5 kidney size, nephron number, and UB tip number were analyzed using One-way ANOVA and Tukey’s test. Graphs were generated using R.

## Electronic supplementary material

Supplementary Information
