# Supplementary material for: Disruption of Hox9,10,11 function results in cellular level lineage infidelity in the kidney
Source: Sci Rep. 2018 Apr 20;8:6306. doi: 10.1038/s41598-018-24782-5 (PMC5910417; doi:10.1038/s41598-018-24782-5)
Supplement: Supplementary file 1 — Supplementary Information [file 41598_2018_24782_MOESM1_ESM.pdf]

Disruption of Hox9,10,11 function results in cellular level lineage infidelity in the kidney

Keri A. Drake[1,2,3], Mike Adam[1], Robert Mahoney[1], and S. Steven Potter*[1]

[1]Division of Developmental Biology, [2]Division of Nephrology and Hypertension, Cincinnati Children's Hospital Medical Center, Cincinnati, OH 45229

[3]Division of Pediatric Nephrology, University of Texas Southwestern Medical Center, Dallas, TX 75390

*Corresponding author:

Steve Potter

Email: Steve.Potter@cchmc.org

Supplemental Table S1. DNA sequence modifications targeted in the *Hoxc9,10,11* mutant mice. The sequences of the *Hoxc9,10,11* targeted alleles after removal of the Kan/Neo gene by Cre recombinase. For the removal of Kan/Neo from the *Hoxc9* and *Hoxc10* genes during the making of the Bac targeting construct Cre expression was arabinose induced in EL350 *E. coli.* The remaining Kan/Neo gene in *Hoxc11* was used for G418 selection of ES cells post electroporation introduction of the BAC targeting construct. The remaining Kan/Neo was then removed from *Hoxc11* by breeding to an *EIIA-Cre* mouse after germ-line transmission of the targeted alleles was established. The net result of targeting for each gene was the insertion of a recombined LoxP sequence (red) into the first exon coding region, as well as a deletion of a coding region from the first exon. In each case the net insertion/deletion resulted in a frameshift mutation, most likely resulting in a null allele. For each targeted allele the green highlighted sequence, and everything 5' of it, as well as the yellow sequence, and everything 3' of it, was wild type. The sequence between green and yellow marks the region of deletion of coding sequence as well as insertion of the recombined, and inactive, LoxP66/71 as well as small regions of flanking DNA that included restriction sites used to make the targeting constructs.

Gene	Sequence of Targeted Allele	Deletion Size
HoxC9	TAAATACGATCACGTGGGGGCTGGGGAACCAATGAGCTGCCGGGAAAAGGCTGGAAAAATAATTACCTGCCTT<br>GATTGTTCTGTGAGCAGATAAAAAGTACATATACAGTTCATACAATAATCTTATGTATGTAAAACCCTGTTACGAT<br>GTCGGCGACGGGGCCCATCAGTAACTATTACGTGGACTCGCTCATCTCTCACGACAATGAAGACCTCCTAGCGTC<br>CAGGTTTCCGGCCACCGGGGCTCACCCTGCCGCCGCCAGACCCAGCGGCTTGGTGCCGGACTGTAGCGATTTTCC<br>GTCCTGGGATCCACCTATACCGTTCGTATAATGTATGCTATACGAACGGTAAAGCTTTCTCCTTCCCCAGCTTCCCG<br>GCCGGGGGCCGTCACTACGCCCTCAAGCCCGACGCCTACCCGGGGCGCCGCGCCGACTGCGGCCCGGGCGACGG	181 bases
HoxC10	TTCCTCCCGCCCCTCCAACCGCTCCCCCCCCCCCTTCCCGGATGGGGGAAAAAAAATGTCAGCTCCTCCGCTGTA<br>GTATTGCTCCTTAAAAACCCCTCTCTNTGAAAATGACATGCCCTCGCAATGTAACTCCGAACTCGTACGCGGAGC<br>CCTTGGCTGCGCCGGGGGGAGGAAAGCGCTATAACCGTAACGCAGGAATGTATATGCAATCTGGGAGTGACTT<br>CAACTGCGGGGGGATAAGCTTTACCGTTCGTATAGCATACATTATACGAACGGTATAGGTGGATCCCGCACCGA<br>ACATCTGGAATCGCCTCAGCTTGGGGGCAAAGTGAGTTTTCCTGAAACCCCCAAGTCCGACAGCCAAACCCCCAG<br>TCCCAATGAAATCAAAACAGAGCAAAGCCTGGCGG	415 bases
HoxC11	AAATTTCCCCCCTCGCTAGATCGGGTCCNAAACCTCCAGCCGGAGGAGGCAGGAGAAGAGAACGATGTTTAACT<br>CGGTCAACCTGGGCAACTTCTGCTCGCCTTCGCGCAAGGAGAGGGGCGCTGACTTCGGCGAACGAGGGAGCTG<br>CACCTCCAACCTCTATCTGCCCAGTTGCACTTACTACGTGCCTGAGTTCTCCACCGTCTCTTCCTGGATCCACCTAT<br>ACCGTTCGTATAATGTATGCTATACGAACGGTAAAGCTTCCCTCTCGTCAGATTTCTTATCCCTACTCAGCCCAAG<br>TGCCCCCGGTCCGGGAGGTCTCCTACGGCCTGGAACCGTCCGGGAAGTGGCACCATCGGAACAGCTACTCGTCT<br>TGTTATGCGGCGGCCGACGAGCTTATGCACCGGGAGTGCCTGCCTCCTTCCACAGTCACTGAGATCCTCATGAAA	58 bases

Blue highlights = ATG start codon
Red highlights = Residual loxP sequences
Yellow highlights = homology block primer
Green highlight = homology block primer

Supplemental Table S2. Primers used to generate *Hoxc9,10,11* BAC targeting construct. These DNA sequences were used to PCR amplify the blocks of homology used for the recombineering of the BAC targeting DNA construct, as described in Fig. 1.

Primers for homology blocks:

HoxC9 (L1)	TCTCctcgagGAAAAAGCAAGGGGGAAAAA
HoxC9 (L2)	TCTCaagcttAGGACGGAAAATCGCTACAG
HoxC9 (R1)	TTCCggatccCCGTCACTACGCCCTCAA
HoxC9 (R2)	CTCTgcggccgcCTCAGACCTTCCTGCTCTTG
HoxC10 (L1)	TCTCctcgagATGCCCTCGCAATGTAACTC
HoxC10 (L2)	TCTCaagcttGGTAGGACGGGTAGGTGTTG
HoxC10 (R1)	TTCCggatccGCCTCTGTCCTCCTGTTCCT
HoxC10 (R2)	CTCTgcggccgcCTGAGGCGATTCCAGATGTT
HoxC11 (L1)	TCTCctcgagTGCTCGGGGAGAGAGACTAA
HoxC11 (L2)	TCTAagcttGGGGTAGGAAGGAAGAGACG
HoxC11 (R1)	TTCCggatccCCATCGGAACAGCTACTCGT
HoxC11 (R2)	CTCTgcggccgcGAGCTGGGATTCGTGTTCTC

Supplemental Figure S1. Successful targeting of embryonic stem cells (ESCs) was verified by counting the wild-type *Hoxc9* alleles. Random integration of the targeting construct leaves two wild type alleles, while targeted recombination replaces one wild type allele with a mutant allele. Genomic DNA was used for the qPCR reactions. Reactions were normalized to the B-actin gene. A relative wild type allele count of 0.5 (arrows) indicated one of the endogenous *Hoxc9* genes was targeted in that ESC clone. qPCR primer/probe sequences used to count the wildtype *Hoxc9* alleles are shown below. Candidate targeted ES clones were expanded and re-verified by another round of qPCR. Final targeted alleles were sequenced for further confirmation. Immunofluorescence showed no detectable Hoxc9 or Hoxc10 protein in the *Hoxc9,10,11* homozygous mutants (data not shown). For Hoxc11 we could not find an appropriate antibody.

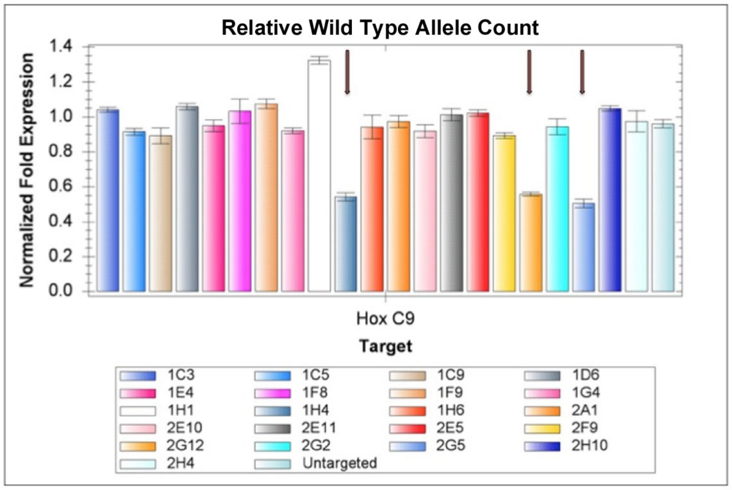

<u>qPCR primers for *Hoxc9*:</u>
- FOR: cggctgtattcagtacgtcg
- PROBE: tcgtctgtggtctatcaccccttacgg
- REV: catctagcgcgtgtcgg

<u>B-Actin-qPCR:</u>
- FOR: agctcaccattcaccatcttg
- PROBE: cctggcctcactgtccacctt
- REV: gactcatcgtactcctgcttg

Kidney Size (E18.5)

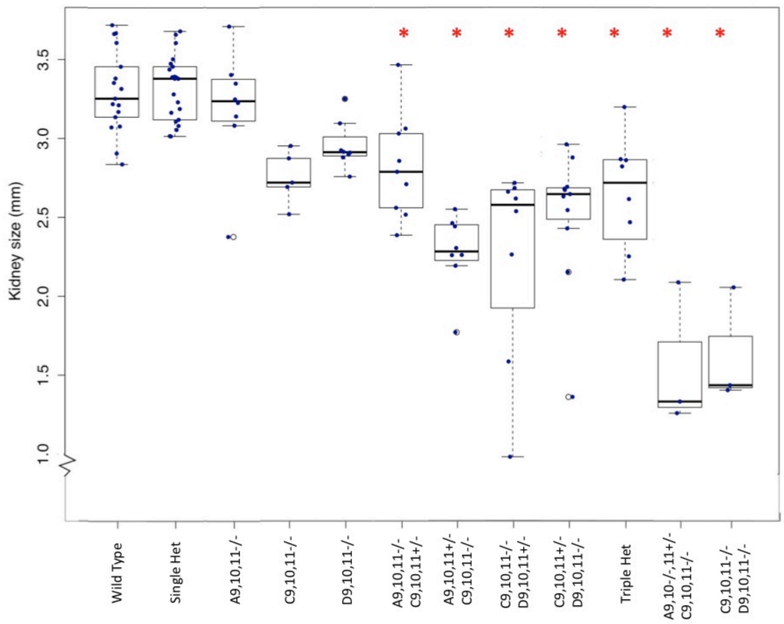

Supplemental Figure S2. Multi-Hox mutant mice showed decreased kidney size at E18.5 (related to Figure 2). Kidney size was quantified (mm) for each genotype (red asterisk – p value < 0.05 vs wild type). Somewhat surprising, even *Hoxc9,10,11-/-* kidneys, with only three flanking Hox genes mutated, and many wild type Hox9,10,11 paralogs remaining, trended smaller (p = 0.067). Each dot represents one mouse.

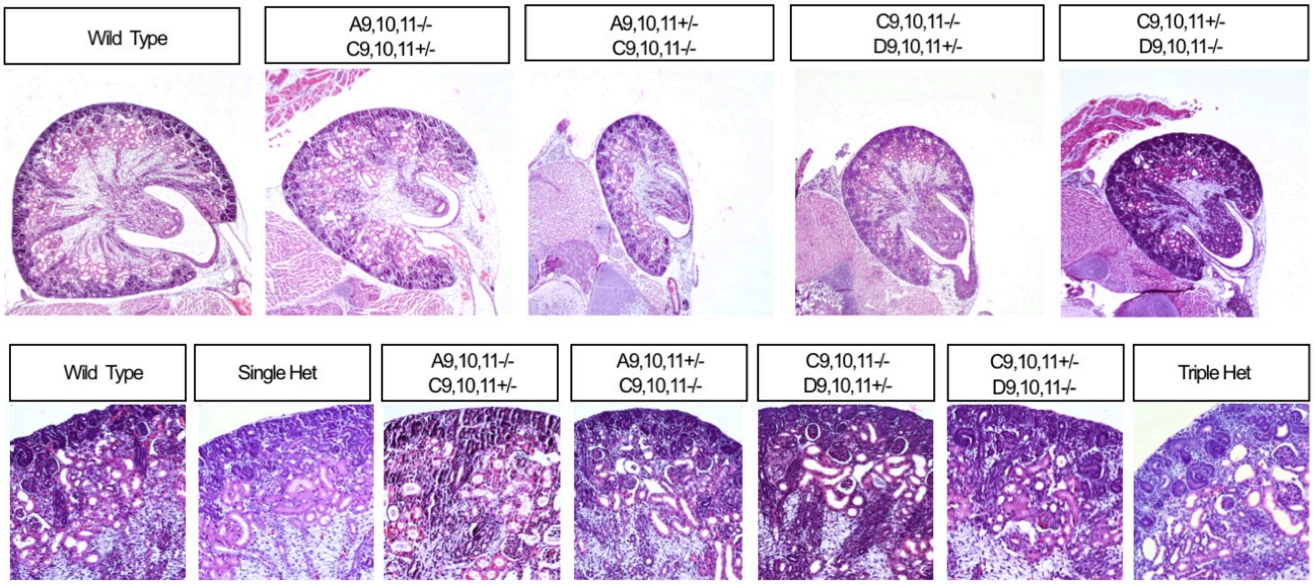

Supplemental Figure S3. Phenotypes of heterozygous/homozygous and triple heterozygous multi-Hox mutant mice did not show distinguishable differences among the different mutant genotypes by histology at E18.5. As described previously, mutli-Hox mutants were small (see Supplementary Fig. S2), but otherwise the genotypes examined (*Hoxa9,10,11-/- Hoxc9,10,11+/-, Hoxa9,10,11+/- Hoxc9,10,11-/-*, *Hoxc9,10,11-/- Hoxd9,10,11+/, Hoxc9,10,11+/- Hoxd9,10,11-/-,* and *Hoxa9,10,11+/- Hoxc9,10,11+/-, Hoxd9,10,11+/-*) were indistinguishable by histology (top row original magnification 4x; bottom row original magnification 20x).

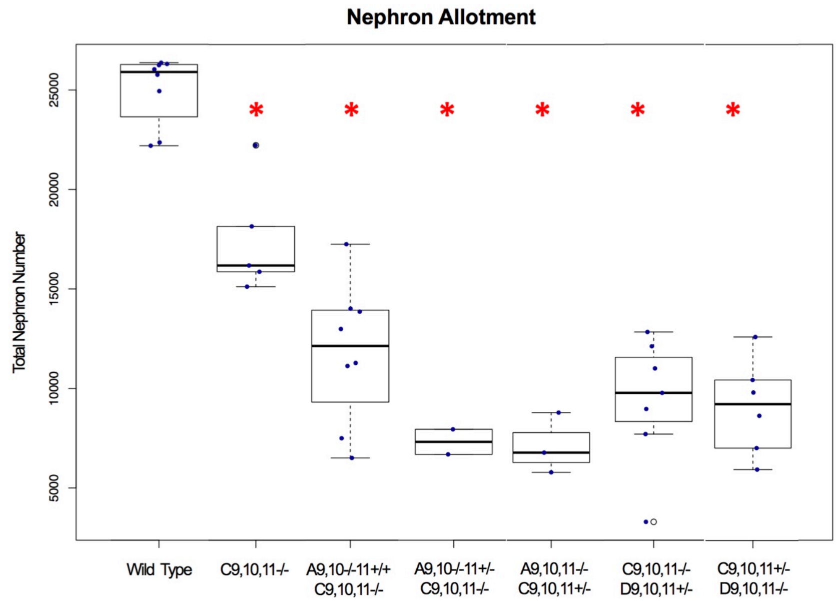

Supplemental Figure S4. Multi-Hox mutant mice showed decreased nephron allotment at P14 (related to Figure 2). Total nephron number was determined using the acid maceration technique to count the number of glomeruli in both kidneys at postnatal day 14. Representative genotypes from heterozygous/homozygous Hox mutant mice (as shown above) demonstrated impaired nephrogenesis, with an approximate 60% reduction in nephron number compared to litter mate wild type controls. Interestingly, mice carrying homozygous mutation of only the three flanking Hoxc9,10,11 genes additionally showed significantly reduced nephron numbers, even though multiple paralogous genes remain intact (each blue dot represents one mouse; red asterisk, $p < 0.05$ vs wild type)

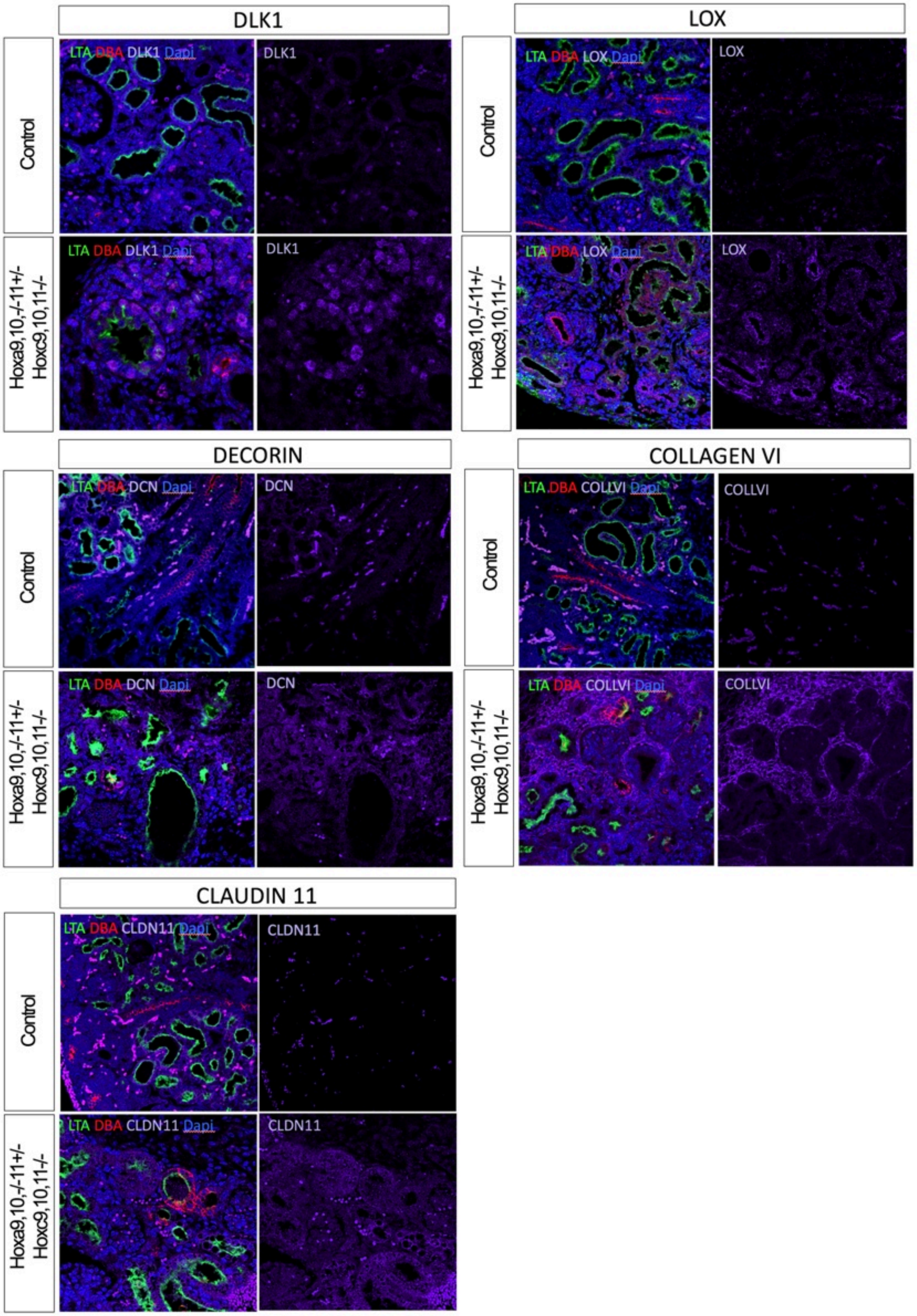
DLK1
Control
LTA DBA DLK1 Dapi
DLK1
Hoxa9,10,-/-11+/- Hoxc9,10,11-/-
LOX
LTA DBA LOX Dapi
LOX
DECORIN
LTA DBA DCN Dapi
DCN
COLLAGEN VI
LTA DBA COLLVI Dapi
COLLVI
CLAUDIN 11
LTA DBA CLDN11 Dapi
CLDN11

Supplemental Figure S5. Multiple upregulated genes from the RNA seq data were validated using immunofluorescence. We validated 5 genes identified from our RNA-seq data (*Dlk1, Lox, Dcn, CollVI,* and *Cldn11*) showing up-regulation in E18.5 Hox mutant kidneys vs. control. N=3 per immunoassay.

Supplemental Table S3. Quantification of asymmetric ureteric bud (UB) branching in Hox mutants (related to Fig. 3a, A'-E').

Genotype		Number Examined	Isolated Dorsal/Ventral Asymmetry	Anterior/Posterior & Dorsal/Ventral Asymmetry	Absent UB Branching
Controls	Wild Type	5	0	0	0
	Single Het	5	0	0	0
Heterozygous/ homozygous	Hoxa9,10,11+/- Hoxc9,10,11-/-	8	25%	75%	0
	Hoxa9,10,11-/- Hoxc9,10,11+/-	8	50%	50%	0
	Hoxc9,10,11+/- Hoxd9,10,11-/-	8	80%	12%	0
	Hoxc9,10,11-/- Hoxd9,10,11+/-	9	55%	45%	0
Double homozygous	Hoxa9,10-/-11+/+ Hoxc9,10,11-/-	3	0	100%	0
	Hoxa9,10,11-/- Hoxc9,10,11-/-	3	0	0	100%
	Hoxc9,10,11-/- Hoxd9,10,11-/-	8	0	88%	12%

Supplemental Table S4. Differential gene expression (RNA-seq) on whole kidneys at E18.5 comparing wild type (N=3) and Hoxa9,10-/-11+/- HoxC-/- (N=2) and Hoxa9,10-/-11+/+ HoxC-/- (N=1) mutants; data filtered on expression level of > 10 RPKM in at least 3 of 12 samples, Audic Claverie analysis, P < 0.05, fold change (FC) > 2.

Gene Symbol	Fold Change [mut] vs [wt]	Regulation [mut] vs [wt]
Dlk1	9.49	up
Lum	8.13	up
Hba-a2	7.49	up
Dcn	4.21	up
Mfap5	3.99	up
Sprr2f	3.94	up
Postn	3.92	up
Col1a1	3.70	up
Twist2	3.67	up
Fbln2	3.52	up
Lyve1	3.48	up
Rdh19	3.43	up
Ogn	3.01	up
Fmo2	2.97	up
Col3a1	2.95	up
Col1a2	2.93	up
Nell2	2.84	up
Lox	2.79	up
Col6a1	2.75	up
Slco1a6	2.72	up
Col6a2	2.71	up
Matn2	2.70	up
Slc5a8	2.66	up
Lgi2	2.65	up
Arsi	2.62	up
Tgm2	2.60	up
Slc3a1	2.58	up
AI317395	2.55	up
Acmsd	2.54	up
Pappa2	2.50	up
Smoc2	2.48	up

Fmod	2.43	up
Gm1821	2.42	up
Slc7a12	2.41	up
Col8a2	2.40	up
Trf	2.37	up
Dpep1	2.36	up
Col14a1	2.35	up
Col5a1	2.34	up
Aspn	2.33	up
G6pc	2.32	up
Olfml3	2.30	up
H2-Ab1	2.28	up
Cldn11	2.27	up
Ace	2.25	up
Cckar	2.25	up
Cd36	2.21	up
Cldn2	2.21	up
Bhmt2	2.19	up
Hsd3b4	2.17	up
Thbs2	2.17	up
Slc22a13	2.17	up
Figf	2.14	up
Slc16a4	2.14	up
Slc6a20b	2.14	up
Hyi	2.13	up
Slc26a1	2.13	up
C1qa	2.13	up
Slc38a3	2.11	up
Kcnj15	2.10	up
Col5a2	2.09	up
Slc5a1	2.09	up
Defb29	2.08	up
Fbp1	2.07	up
Adm	2.07	up
Aoc1	2.07	up
Slc22a6	2.05	up
Mogat2	2.05	up
Eln	2.05	up
Igfbp6	2.03	up
Slc5a11	2.03	up
Cyp2d26	2.02	up

Glo1	2.02	up
Sult1c2	2.01	up
Stab1	2.01	up
Slc22a1	2.00	up
Aldh1a3	-2.01	down
Mycn	-2.02	down
Crym	-2.03	down
Mki67	-2.05	down
Leng8	-2.06	down
Etv4	-2.08	down
Wnt11	-2.09	down
Gadd45g	-2.10	down
Tmem100	-2.10	down
Srsf4	-2.14	down
Ccnl2	-2.25	down
Snhg1	-2.29	down
Spink8	-2.38	down
Col2a1	-2.39	down
Scx	-2.41	down
Clk1	-2.44	down
Wsb1	-2.46	down
Snhg5	-2.52	down
Dkk1	-2.85	down
Cited1	-2.95	down
Ccnl1	-2.96	down
Eya1	-2.98	down
Six2	-3.00	down
Malat1	-3.00	down
Snora65	-3.01	down
Rprm	-3.07	down
Psca	-3.19	down
Gdnf	-3.45	down
Clec2d	-4.08	down
Erdr1	-4.86	down
Mir3064	-7.71	down

Supplemental Table S5. Primary/secondary antibody and lectin list

Antibody	Species	Source	Dilution
Six2	Rabbit	Proteintech; 11562-1-AP	1:200
Calbindin-D-28K	Mouse	Sigma; C9848	1:200
Hnf4a	Rabbit	Santa Cruz; sc-8987	1:50
Hnf4a	Goat	Santa Cruz; sc-6556<br>(gift from Ondine Cleaver)	1:50
Slc12a1	Rabbit	Proteintech; 18970-1-AP	1:200
Cytokeratin – 1,5,6,8	Mouse	Sigma; C1801	1:200
Slc8a1	Rabbit	Abgent; AP8939c-ev	1:100
Villin	Rabbit	Abcam; ab130751	1:100
Villin	Mouse	Santa Cruz; sc-58897	1:100
Ecad	Rat	Sigma; U3254	1:200
Dlk1	Rabbit	Proteintech; 10636-1-AP	1:200
Lox	Rabbit	Abcam; ab174316	1:200
Decorin	Rabbit	Santa Cruz; sc-22753	1:200
CollVI	Rabbit	Abcam; ab182744	1:200
Cldn11	Rabbit	Proteintech; 12152-1-AP	1:200

Lectin	Conjugated Label	Source	Dilution
Lotus tetragonolobus (LTA)	Fluorescein (495/515)	Vector labs; FL-1321	1:100
Dolichus Biflorus Agglutinin (DBA)	Rhodamine	Vector labs; RL-1082	1:100
Dolichus Biflorus Agglutinin (DBA)	Streptavidin	Vector labs; B-1035	1:100

Secondary Antibody	Species	Source	Dilution
Rabbit - 488	Chicken	ThermoFisher; A-21441	1:500
Rabbit - 488	Donkey	ThermoFisher; A-21206	1:500
Rabbit - 546	Goat	ThermoFisher; A-11035	1:500
Rabbit - 647	Donkey	ThermoFisher; A-31573	1:500
Goat - 488	Donkey	ThermoFischer; A-11055	1:500
Mouse - 568	Donkey	ThermoFischer; A-10037	1:500
Mouse IgG1 - 633	Goat	ThermoFisher; A-21126	1:500
Streptavidin - 488	N/A	ThermoFisher; S-32354	1:500
Streptavidin - 647	N/A	ThermoFisher; S-21375	1:500
